# Reliability and Validity Testing of the Persian Version of the Derriford Appearance Scale 24 in a Sample of Individuals with Craniofacial Irregularity and Amputation

**DOI:** 10.33137/cpoj.v6i1.41454

**Published:** 2023-11-24

**Authors:** A Khani, T Babaee, A Khaghani, M Nakhaee, Z Fatahi, T Moss

**Affiliations:** 1 Department of Orthotics and Prosthetics, University of Social Welfare and Rehabilitation Sciences, Tehran, Iran.; 2 Rehabilitation Research Center, Department of Orthotics and Prosthetics, School of Rehabilitation Sciences, Iran University of Medical Sciences, Tehran, Iran.; 3 Department of Rehabilitation, Faculty of Allied Medicine, Kerman University of Medical Sciences, Kerman, Iran.; 4 Centre for Appearance Research, University of the West of England, Bristol, UK.

**Keywords:** Appearance, Disfigurement, Craniofacial defect, Amputation, Prosthesis, Rehabilitation

## Abstract

**BACKGROUND:**

Despite the recent advancements in the design and manufacture of prostheses for individuals with craniofacial irregularity and amputation, these individuals tend to become self-conscious about their appearance. The aim of this study was to investigate the reliability and validity of Persian version of the Derriford Appearance Scale24 (P-DAS24) for a sample of individuals with craniofacial irregularity and limb loss.

**METHODOLOGY:**

Reliability of the P-DAS24 was determined by computing internal consistency and test-retest reliability utilizing Cronbach's alpha coefficient and Pearson's correlation coefficient. Discriminant validity was investigated with comparing the total score of the P-DAS24 between disfigured participants and those with no appearance problem. Known-groups validity was evaluated regarding the participants' gender and their level of involvement.

**FINDINGS:**

The sample size comprised of 251 individuals with disfigurement and 101 without disfigurement who were deemed normal in appearance. The P-DAS24 showed satisfactory internal consistency (Cronbach's alpha = 0.89) and excellent test-retest reliability (r = 0.96). The total score of the P-DAS24 showed a statistically significant difference between individuals deemed disfigured or normal (P=0.01). The total scores P-DAS24 in individuals with different levels of involvement were significantly different (P<0.001). The scores of the DAS2, DAS18, DAS21, and DAS24 were significantly different between men and women (P<0.01, <0.01, 0.03, and 0.01, respectively).

**CONCLUSION:**

The P-DAS24 is a valid and reliable tool that may be utilized in clinical practice and researches to assess the outcomes of prosthetic reconstructions in individuals with disfigurement.

## INTRODUCTION

Disfigurement can be defined as a visible difference or unusual appearance as a result of a mark, rash, scar, or graft on a person's skin or an asymmetry or paralysis to a person's face or body.^1^ Disfigurement might occur due to congenital malformation, traumatic events, and disease processes.^2^ Traumatic amputation, amputation due to Diabetes Mellitus and burn injuries are among the leading causes of disfigurement in low to middle-income countries.^3,4^ Despite the recent advancements in the design and manufacture of prostheses to improve their natural size, shape, and movement, prosthesis users may become self-conscious about their appearance.^5^ This is partly due to the judgment and stigma they experience in their daily life.^6^

The extent to which the disfigurement affects one's perception of his/her appearance depends on two main factors.^7^ The first factor which is mainly social and cultural, which include social cues and feedback one might get from others. Disfigured individuals are usually less involved in social activities and tend to be isolated from society. The second factor is the individual's self-perception determined by the impact of appearance on one's perception of self-concept, emotional well-being, and quality of life. Being different from others might lead to feelings of shame, lower self-esteem, and appearance consciousness.^7,8^

Facially or physically disfigured individuals encounter more psychosocial problems in their daily lives than individuals deemed attractive.^9^ Social stereotypes have led to the general assumption that physically attractive adults have more socially appealing personalities and live more fulfilling lives than those of lesser attractiveness.^10^ Furthermore, they receive more attention and social support in childhood and adulthood.^11^ On the other hand, disfigured individuals experience anxiety, discomfort, and alienation from other members of society.^12^

Psychological adjustment to the injury and disfigurement is a challenging phase when returning to society^7^ as social discomfort and body image anxiety is relatively higher in some people with amputation than individuals without amputation.^13^ Based on data of a study published in 2021, a total of 57.7 million people were living with limb loss globally.^14^ Therefore, it is of paramount importance for the therapist/clinician to understand the psychosocial factors affecting one's adaptation process to disfigurement/amputation to adopt the most effective therapeutic approach and navigate a better restoration of body identity.^15^ Various psychometric tools have been developed to assess the psychological impact and adjustment of appearances such as the Appearance Schemas Inventory,^15^ the Body Image Avoidance Questionnaire,^16^ and the Body Dysmorphic Disorder Examination.^17^ These questionnaires lack the necessary sensitivity to the nature of the irregularity and were not specifically designed to fully cover the spectrum of symptoms relevant to the wide range of difficulties experienced by individuals with disfigurement.^12^ Given the complexity of how an appearance difference might affect one's social interaction and the resulting distress, the measure for this assessment should be well-standardized and psychometrically robust in order to fully capture the distress and dysfunction arising from body-image disturbance.^18^

The Derriford Appearance Scale (DAS) is a valid and factorial scale that examines the psychosocial adjustment issues of subjects with a visible difference.^18^ This questionnaire examines the negative emotions of people with facial problems (such as fear, social anxiety, and shyness) and negative behaviors such as isolationism that may affect their lifestyle.^19^ DAS is available in two versions: DAS59 and the short form of DAS24. DAS24 specifically assesses appearance-related distress and dysfunction. In a study of 525 individuals with appearance problems, the DAS24 total score showed excellent internal consistency and concurrent validity with the DAS59.^19^ The DAS24 questionnaire is suitable for patients on the plastic surgery list, people with cutaneous acne, neurofibromatosis, and eczema and it has formerly been used in a wide range of individuals who had appearance-related issues in different parts of their body.^20^ The questionnaire has also been widely used to assess general population facial concerns.^20^ Although the Persian version of DAS59 was made available by Sadeghi-Bazargani et al.,^9^ the lengthy nature of the DAS59 has made its use time-consuming. Routine data collection in clinical practice and measurement of distress when there is a severe lack of time necessitates validation of its short form with the same sensitivity and improved time efficiency. The robust nature of DAS24 has made this questionnaire a more user-friendly and brief instrument for clinicians to evaluate the therapeutic outcome of the treatment process of individuals with disfigurement.^17^

The DAS24 has so far been translated into Taiwanese,^21^ Brazilian/Portuguese^22^ and Italian.^23^ It should be noted that both versions of DAS59 and DAS24 are available in Italian language.^23,24^ Given the ethnic, linguistic, and cultural differences between different communities that can influence how patient-centered questionnaires are completed, it is necessary to conceptually translate and validate the text of the questionnaire and confirm its reliability and validity to use scales such as DAS24. Also, in order to improve the generalizability and proper use of this instrument for individuals using cosmetic or functional prosthesis, this study was aimed at providing the Persian version of the DAS24 for a sample of individuals with craniofacial irregularity and limb loss.

## METHODOLOGY

### Translation and Cultural Adaptation

**Figure 1:**
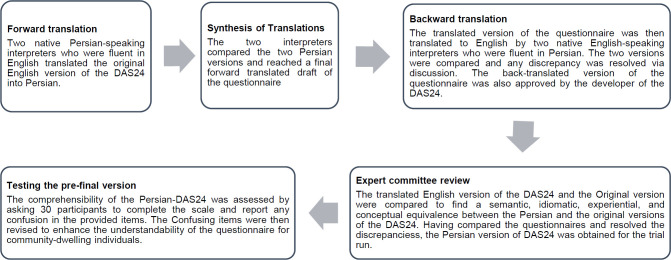
The cultural adaptation process of the Persian-DAS24.

The process of cultural adaptation of the DAS24 was conducted using the guidelines introduced by Beaton et al.^25^ There were five main stages of forward translation, synthesizing of translations, backward translation, expert committee revision and the pre-final trial run (**Figure 1**). Prior to initiation of the study, permission to translate and culturally adapt the DAS24 was obtained from the developer via email. The study protocol was approved by the research ethics committee of Kerman University of Medical Sciences (Ref: # IR.KMU.REC.1399.413, date: 27/07/1399). The final Persian version of the DAS24 is available as a supplementary file.

### Participants and Sampling

A convenience sample of 352 participants were enrolled in this study. 251 of whom had a disfigurement at some level and 101 individuals were normal and not visibly different. The disfigurement group had a discernible difference either in their appearance or daily function with the prosthesis. The inclusion criteria for the disfigurement group included: 1) being 18 or older, 2) having had facial amputation or limb loss with detectable dissimilarity, 3) having used a prosthesis for at least 3 months, 4) being able to read and write in Persian.

The research was conducted in two Orthotics and Prosthetics clinics which were referral centers for patients from all over Iran. Data collection was carried out from November 2021 to August 2022. The sample included those who had lost their body parts due to trauma (accidents, war), cancer, diabetes, burn, vascular disease or the cause of their disfigurement was congenital. The survey was filled either in person or online. Before filling in the questionnaire, subjects were provided a consent form to sign. For the online version, an invitation message was sent via WhatsApp which included a brief description of the study and a link to the questionnaire. Consent was obtained through the inclusion of a statement embedded at the beginning of the online survey. The phone number of the respondents were automatically removed by the software used (https://porsline.ir). The participants remained anonymous through the statistical analyses.

### The Derriford Appearance Scale 24

The DAS24 is a twenty-four-item instrument aimed to assess the anxiety and impairments that results from abnormalities in one's appearance.^26^ The first page of the scale consists of two parts. The upper section includes the demographic information, and the lower section determines the body part(s) that has disturbed the participants' appearance. The primary section of the questionnaire includes 24 items. Some of the items are presented with 4 and some are presented with 5 responses. For items a, b, d, h, j, n, q, t, and v the scores range from 1 (minimum discomfort) to 4 (maximum discomfort). The remaining 14 items are given scores from 0 (not applicable) to 4 (maximum discomfort). Thus, the minimum score for a respondent in DAS24 is 11 and the maximum is 96. The greater the score, the more self-conscious one feels about his/her appearance.

### Data analyses Reliability

Reliability of the Persian version of DAS24 (P-DAS24) were assessed by computing internal consistency and also test-retest reliability. Internal consistency or homogeneity of a scale represent the relationship between the score of each individual item and the total score.^27^ Test-retest reliability is to assess the reproducibility of the same scores for a scale over repeated trials. To investigate the test-retest reliability of the P-DAS24, a sample of 39 participants (19 cases with transtibial amputation, 9 with transfemoral amputation, 7 with craniofacial irregularity, 3 with Syme disarticulation, and 1 with knee disarticulation) were randomly asked to complete the scale once again with the time interval of 2 weeks. For internal consistency assessment of the P-DAS24, Cronbach's alpha coefficient was used. Satisfactory internal consistency is determined by a value of 0.70 ≤ alpha ≤ 0.95 for Cronbach's alpha.^28^ To investigate the relationship between respective item of the P-DAS24 with the entire scale, the values of “Corrected Item-total Correlation” was evaluated. The value of higher than 0.3 was deemed that each item has an agreeable relationship with the entire questionnaire.^28^ “Cronbach's alpha if item deleted” was run to verify whether the extent of the Cronbach's alpha increased since eliminating one item of the questionnaire. To assess the test retest reliability, the Pearson correlation coefficient analysis was used.

### Discriminant validity

Discriminant validity is known as the ability of an instrument to differentiate between individuals with disfigurement and the normal participants.^27^ In this study, discriminant validity of P-DAS24 was assessed by conducting a comparison of the P-DAS24 total score between disfigured participants and those with no appearance problem using Mann-Whitney U test.

### Known-groups Validity

In this technique, an instrument is deemed valid if it shows different scores for groups which are known to have different characteristics.^27^ We assessed the known-groups validity of the P-DAS24 regarding the gender of participants and their level of involvement (maxillofacial, upper limb amputation, transtibial amputation, knee disarticulation, transfemoral amputation, and partial foot amputation/ankle disarticulation using Kruskal-Wallis analysis of variance. Previous studies have shown that the gender and site of involvement affects individual's response to the DAS24 questionnaire.^19,21,22^ For the purpose of this study and to gain more reliable statistical results, individuals with involvements at eye, ear, nose and jaw were grouped as maxillofacial. Also, the amputees with partial foot and rear-foot amputations were grouped as partial foot amputation/ankle disarticulation. Moreover, those with transtibial amputation, knee disarticulation, and transfemoral amputation were categorized in their own group.

Descriptive statistical information was reported by mean and standard deviation (SD). The relationship between the P-DAS24 total score and time since amputation and time using prosthesis was investigated using Spearman correlation coefficient.

A threshold of 0.05 was considered as the statistical significance level with 95% confidence intervals for all correlation coefficients. All statistical analysis was conducted using the SPSS software program version 24 (SPSS Inc., USA).

## RESULTS

### Participants

The sample size comprised of 251 individuals with disfigurement and 101 without disfigurement who were deemed normal in appearance. The average age of the participants was 38.49 years old.

Of the disfigured individuals, 201 (80%) were men. War-related traumatic amputation and accidents were the leading causes of disfigurement accounting for 37.8% and 35.1% of the disfigured population.

Participants' level of disfigurement was seen at different levels with transtibial amputation (44.2%) being the first followed by transfemoral amputation (18%), maxillofacial irregularities (11.9%), Syme amputation (11.5%), upper limb amputation (10%) and knee disarticulation (4.4%). The demographics of participants is revealed in (**Table 1**).

**Table 1:** Demographic and clinical characteristics of the studied population (n=251).

Variables	N (%)
Cause of problem	
Diabetes	16 (6.4)
Accidents	88 (35.1)
War-related traumatic amputation	95 (37.8)
Cancer	11 (4.4)
Congenital	18 (7.2)
Burn	23 (9.2)
Level of appearance problem	
Maxillofacial	30 (11.9)
Upper limb amputation	25 (10.0)
Transfemoral	45 (18.0)
Knee disarticulation	11 (4.4)
Transtibial	111 (44.2)
Partial foot and ankle amputation	29 (11.5)
Occupational status	
Working	191 (76.1)
Unemployed	21 (8.3)
Student	6 (2.4)
Homemaker	28 (11.1)
Missing	5 (2.0)
Family status	
Married	177 (70.5)
Living with relatives	34 (13.5)
Living alone	37 (14.7)
Missing	3 (1.2)
Educational status	
High school	50 (19.9)
Diploma	99 (39.4)
Bachelor	56 (22.3)
Master	34 (13.5)
PhD	10 (4.0)
Missing	2 (0.8)

### Reliability

The P-DAS24 demonstrated excellent internal consistency (Cronbach's alpha = 0.89). Regarding corrected item-total correlation, our results showed that items DAS8, DAS10, and DAS15 had a value < 0.3. Still, the Cronbach's alpha if item deleted was exceeding 0.7 for the whole P-DAS24. Cronbach's alpha if item deleted and Corrected Item Total Correlation scores confirmed that removing an item will not improve the value of Cronbach's alpha, therefore all of the items were maintained (**Table 2**). The scale total score of the P-DAS24 showed excellent test-retest reliability (r=0.96) (**Table 2**).

**Table 2:** Descriptive statistics and results of the reliability analyses: test-retest reliability (n=39) and internal consistency (n=251). Abbreviations: DAS = Derriford appearance scale; SD = standard deviation.

DAS24 Items	Mean (SD)	Corrected Item-Total Correlation	Cronbach's Alpha If Item Deleted	Pearson's correlation coefficient for test-retest reliability
DAS1	1.77 (0.84)	0.50	0.73	0.90 (0.82–0.94)
DAS2	1.90 (1.08)	0.63	0.73	0.99 (0.98–0.99)
DAS3	1.04 (1.05)	0.65	0.73	0.91 (0.84–0.95)
DAS4	2.99 (1.04)	0.51	0.73	0.93 (0.87–0.96)
DAS5	1.24 (1.15)	0.65	0.73	0.93 (0.87–0.96)
DAS6	1.76 (1.44)	0.50	0.73	0.97 (0.95–0.98)
DAS7	1.57 (1.17)	0.43	0.73	0.94 (0.89–0.96)
DAS8	2.21 (1.23)	0.19	0.74	0.88 (0.79–0.94)
DAS9	1.07 (1.08)	0.63	0.73	0.90 (0.82–0.95)
DAS10	2.53 (0.96)	0.19	0.74	0.86 (0.75–0.92)
DAS11	1.30 (0.64)	0.34	0.74	0.88 (0.78–0.93)
DAS12	1.76 (1.41)	0.59	0.72	0.94 (0.89–0.97)
DAS13	1.12 (0.98)	0.48	0.73	0.98 (0.96–0.99)
DAS14	1.43 (0.80)	0.59	0.73	0.91 (0.83–0.95)
DAS15	0.70 (0.85)	0.22	0.74	0.84 (0.71–0.91)
DAS16	1.96 (1.30)	0.58	0.73	0.90 (0.82–0.94)
DAS17	2.64 (1.23)	0.54	0.73	0.94 (0.89–0.97)
DAS18	2.08 (1.36)	0.63	0.72	0.83 (0.70–0.91)
DAS19	1.44 (1.09)	0.69	0.73	0.95 (0.92–0.97)
DAS20	2.07 (0.98)	0.51	0.73	0.88 (0.79–0.94)
DAS21	1.01 (1.09)	0.57	0.73	0.92 (0.85–0.96)
DAS22	1.11 (0.89)	0.43	0.73	0.75 (0.57–0.86)
DAS23	1.72 (1.23)	0.56	0.73	0.95 (0.91–0.97)
DAS24	1.25 (1.07)	0.55	0.73	0.80 (0.66–0.89)
**Total**	**39.80 (14.26)**	**1.00**	**0.89**	**0.96 (0.93–0.98)**

### Discriminant Validity

The total score of the P-DAS24 showed a statistically significant difference between individuals deemed disfigured or normal (P=0.01).

However, scores of items DAS2, DAS3, DAS5, DAS9, DAS10, DAS11, DAS13, DAS14, DAS15, DAS18, DAS21, DAS23, DAS24 did not appear to have a significant difference between the two groups (P>0.05, 0.55, 0.10, 0.97, 0.06, 0.44, 0.64 0.08, 0.57, 0.14, 0.25, 0.65, 0.35, respectively) (**Table 3**).

**Table 3:** Known-groups validity of the Persian-DAS24.

Items	Sex		Level of amputation						Normal and Disfigured individuals	
	Male (n=201)	Female (n=50)	Maxillof acial (n=30)	Upper Limb Amputation (n=25)	Transfemoral (n=45)	Knee (n=11)	Transtibial (n=111)	Partial foot amputation and ankle disarticulation (n=29)	Normal Individuals (n=101)	Disfigured Individuals (n=251)
**DAS1**	123.63	133.36	182.17	166.78	113.99	113.95	111.21	112.57	208.75	163.52
***p***	0.36		0.00						0.00	
**DAS2**	119.25	149.71	203.73	169.54	105.17	104.64	112.94	93.71	160.08	181.67
***p***	0.00		0.00						0.05	
**DAS3**	122.64	132.26	199.92	142.35	123.00	149.36	107.04	95.67	179.84	173.06
***p***	0.37		0.00						0.55	
**DAS4**	123.69	133.11	186.57	137.12	126.17	80.18	114.64	114.38	105.07	205.24
***p***	0.38		0.00						0.00	
**DAS5**	127.72	113.60	196.70	147.26	131.05	139.32	105.56	95.74	188.84	170.17
***p***	0.20		0.00						0.10	
**DAS6**	127.16	110.56	176.12	108.43	127.41	146.32	122.77	77.10	149.14	185.53
***p***	0.14		0.00						0.00	
**DAS7**	126.97	116.54	177.87	124.44	115.94	130.73	119.27	108.71	156.51	183.87
***p***	0.35		0.00						0.01	
**DAS8**	125.27	108.38	129.65	125.61	129.52	110.23	118.32	121.79	151.72	181.02
***p***	0.12		0.89						0.00	
**DAS9**	122.62	137.61	194.78	161.92	123.93	126.59	106.61	101.09	176.20	176.62
***p***	0.17		0.00						0.97	
**DAS10**	121.94	127.48	148.15	114.52	117.15	182.23	122.68	94.17	188.64	167.35
***p***	0.61		0.0						0.06	
**DAS11**	121.97	127.46	164.43	138.37	119.35	94.50	114.84	119.36	168.74	175.44
***p***	0.52		0.00						0.44	
**DAS12**	121.30	135.19	188.47	136.70	123.66	91.55	113.70	104.74	158.18	181.85
***p***	0.21		0.00						0.04	
**DAS13**	128.02	114.89	179.62	133.16	117.08	123.77	122.99	90.57	173.00	177.91
***p***	0.21		0.00						0.64	
**DAS14**	124.32	127.84	187.73	137.78	127.70	120.77	111.51	101.98	187.81	171.23
***p***	0.69		0.00						0.08	
**DAS15**	125.43	115.32	140.02	119.87	128.28	118.23	123.14	109.66	178.33	172.25
***p***	0.33		0.60						0.57	
**DAS16**	123.36	134.51	191.38	140.52	119.60	80.73	118.76	100.67	142.78	189.24
***p***	0.32		0.00						0.00	
**DAS17**	124.73	128.74	185.92	147.06	123.48	103.73	113.18	107.28	129.29	194.61
***p***	0.71		0.00						0.00	
**DAS18**	119.11	149.68	178.53	132.78	125.81	120.45	118.53	92.24	163.99	180.85
***p***	0.00		0.00						0.14	
**DAS19**	124.73	128.73	203.63	142.58	116.37	119.68	111.08	105.84	164.72	180.49
***p***	0.71		0.00						0.16	
**DAS20**	124.81	128.41	177.25	138.36	140.67	71.59	114.32	104.91	143.61	189.74
***p***	0.74		0.00						0.00	
**DAS21**	128.28	105.80	181.62	121.89	127.32	104.86	114.14	110.29	165.43	178.16
***p***	0.03		0.00						0.25	
**DAS22**	124.47	127.23	168.67	148.73	108.73	137.18	112.42	133.26	199.63	166.45
***p***	0.78		0.00						0.00	
**DAS23**	124.16	131.13	197.32	153.92	120.06	137.27	102.69	122.31	180.20	175.01
***p***	0.53		0.00						0.65	
**DAS24**	130.41	104.82	185.02	144.46	122.08	146.41	105.93	124.19	169.42	179.35
***p***	0.01		0.00						0.35	
**Total**	124.57	129.40	123.48	153.06	124.00	118.41	106.18	83.66	156.36	184.61
***p***	0.67		0.00						0.01	

### Known-groups Validity

The total scores of DAS24 for individuals with different levels of involvement were significantly different (P<0.001). Also, the scores of each item in P-DAS24 were shown to be significantly different with regard to the level of involvement except for DAS8 and DAS15 (P>0.89 and >0.60, respectively). Although the total score did not indicate a statistically significant difference regarding the gender of the individuals (P=0.67), the scores of the DAS2, DAS18, DAS21, and DAS24 were significantly different between men and women (P<0.01, <0.01, 0.03, and 0.01, respectively) (**Table 3**).

The results of Spearman Correlation Coefficient analysis revealed no statistically significant relationship between time since amputation, time using prosthesis and DAS total score (P>0.05).

## DISCUSSION

This study was set out to validate and cross-culturally adapt the Persian translated version of the DAS24 in a sample of individuals with upper and lower limb amputations and maxillofacial irregularities. Overall, the P-DAS24 demonstrated satisfactory reliability. Therefore, the P-DAS24 is a valid and reliable tool for the clinical evaluation of the appearance-related distress, anxiety and self-consciousness experienced by individuals with visual difference on a daily basis in Persian speaking countries.

The DAS24 is a unidimensional scale extracted from the DAS59 which generates a full-scale score in five factorial subscales of general self-consciousness of appearance, social self-consciousness of appearance, negative self-concept, sexual and bodily self-consciousness of appearance and facial self-consciousness of appearance.^11,19^

The total score of the P-DAS24 showed acceptable internal consistency as reflected by the Cronbach's alpha which was 0.89. This was similar to the internal consistency of the Original English version,^19^ the Brazilian/Portuguese,^22^ and the Italian version^23^ of the DAS24 (Cronbach's alpha= 0.92, 0.94, 0.93, respectively). Our results were similar to that of the Persian DAS59 which revealed excellent internal consistency (Cronbach's alpha = 0.93).^9^ In that study, participants had suffered a burn injury in the face, head, ear, neck, hand and legs, however, the individuals in our study had disfigurements due to amputation. Regarding the item-total correlation, the statistical analysis revealed that items 8, 10, and 15 of the P-DAS24 were less than the cut-off value of 0.3. However, as confirmed by the results of Cronbach's alpha if items are deleted, all P-DAS24 items were associated with the entire scale (Cronbach's alpha > 0.7).

However, Moss et al. concluded that by removing 5 items of the Taiwanese version of DAS24, the Cronbach's alpha of the instrument would increase to 0.97 and therefore, the Taiwanese version includes only 19 items.^21^ The present study also revealed that the P-DAS24 demonstrated excellent test-retest reliability (r= 0.96) which was similar to the findings of the original English and the Taiwanese versions (r= 0.82, 0.88, respectively).^19,21^

As to assess the known-groups construct validity, we compared the means and total scores of the P-DAS24 in individuals with regard to their gender and level of involvement. In terms of gender, the total score of P-DAS24 did not show a significant difference between men and women. This is in contrast to the notable difference between men and women in the Original English^19^ and Brazilian/Portuguese versions^22^ This might be due to the fact that Iranian women are supposed to wear modesty dressings known as ‘Hijab’ which prevents them from exposing their body in the society. However, in items DAS2 ‘Distressed at reflection’ and DAS18 ‘Distressed by clothing limitations’, in which certain amounts of exposure were required and women are stereotypically expected to maintain their feminine physique,^29^ women were reported to experience significantly higher distress than men. This was in line with the findings of a recent study revealing that body image perception was significantly different between men and women with lower limb amputation^30^ and women were more affected than men in relation to body image anxiety.^31^ However, significantly higher scores of men for item DAS24 ‘Avoid restaurants’ revealed the higher distress experienced by them in a social occasion.

Women, on the other hand, had a significantly lower score for this item, which can be explained by the cultural difference between women's dressing in an Islamic community to that of a western community and how easier for Muslim women is to conceal their body parts. One limitation of this study was the heterogeneity of the included participants regarding their cause of irregularity. The DAS-24 has been developed to assess distress and dysfunction to problems of appearance in individuals with any types of visible irregularity. Therefore, this questionnaire has been widely used in different sample of participants with minimal to severe forms of disfigurement. In other translated versions of this questionnaire, the study samples composed of heterogeneous individuals with visible irregularities including burn, cleft lip/palate, hemangioma, neurofibromatosis, oral cancer, and HIV/AIDS.^21,22^ That's why we included individuals with different cause of disfigurement.

Facially or physically disfigured individuals encounter various psychosocial problems depending on the site and level of their amputation or disfigurement.^6^ Our findings indicate that the total score of individuals with different disfigurements were significantly different in P-DAS24. Similarly, Moss et al. reported a significant effect of the body site affected on the scoring of participants in their study (P<0.0001).^19^ Depression and anxiety seen in upper limb amputees are significantly higher than that of lower limb amputees.^31^ Among lower-limb amputees, the individuals with transtibial amputation can conceal their difference/irregularity more easily than transfemoral amputees and hence experience less scrutiny in the society.^5,32^

Prosthetic reconstruction has proved to improve the self-image and self-esteem of those missing a body part.^33^ This might be due to the fact that by means of various prosthetic designs and the advancement in their aesthetic appearance, disfigured individuals are able to conceal their difference more easily and thus avoid the stigma and distress they encounter in social occasions. The DAS24 has previously been used to determine changes in the distress level of patients undergoing rhinoplasty and the results showed a significant decrease in their stress level after the surgery.^34^ However, since it takes a considerable amount of time for amputees to adapt themselves to their prostheses and the cross-sectional nature of our study design, we were not able to compare the self-image perception and body image anxiety of amputees before and after the prosthetic reconstruction. We recommend future studies focus on the impact of prosthetic reconstruction on self-image disturbance of individuals with maxillofacial irregularity and limb loss.

In terms of discriminant validity, our findings revealed that the P-DAS24 is able to discriminate between the normal population and the group with disfigurement (P=0.01). Visible dissimilarity exposes individuals to social isolation that might occur as a result of the timidity, anxiety and distress they experience regularly.^21^ In a sample of Taiwanese individuals, it was shown that the visibly different group scored significantly higher scores for DAS24 compared to the non-visibly different group.^21^ Also, the mean scores of the general population and the clinical population participating in the study by Moss et al. were significantly different (P<0.0005).^19^ Although participants in our study were all using prosthesis as to compensate for the functional and cosmetic aspect of their body, the results showed significantly higher incidence of distress and anxiety in these individuals. This shows the sensitivity of this construct to underlying conditions leading to visible difference and its impact on social interaction of people with disfigurements.

## CONCLUSION

This study provided an effective translation and culturally adapted version of the DAS24 to assess the distress and self-image disturbance of disfigured individuals. This instrument can be used in clinical practice and researches to assess the outcomes of prosthetic reconstructions in individuals with disfigurement.

## DECLARATION OF CONFLICTING INTERESTS

The authors declare that there is no conflict of interest.

## AUTHORS CONTRIBUTION

- **Alireza Khani and Taher Babaee:** writing (original draft preparation, review and editing), design and conceptualization.
- **Alireza Khaghani, Masoomeh Nakhaee, Timothy Peter Moss:** design and conceptualization, writing (review and editing).

All authors have read and agreed to the published version of the manuscript.

## SOURCES OF SUPPORT

This work was supported by the research ethics committee of Kerman University of Medical Sciences.

## ETHICAL APPROVAL

The study protocol was approved by the research ethics committee of Kerman University of Medical Sciences (Ref: # IR.KMU.REC.1399.413, date: 27/07/1399).
